# Iron Deficiency Without Anaemia in a Young Female With Idiopathic Intracranial Hypertension: A Case Report Supporting Routine Iron Studies

**DOI:** 10.7759/cureus.93334

**Published:** 2025-09-27

**Authors:** Rahul Premarajan

**Affiliations:** 1 Acute Medicine, Great Western Hospital, Swindon, GBR

**Keywords:** case report, idiopathic intracranial hypertension, iron deficiency, papilledema, polycystic ovary syndrome, pulsatile tinnitus

## Abstract

Idiopathic intracranial hypertension (IIH) is common in young, overweight women, but less recognised factors - such as iron deficiency - may also play a role. We report the case of a 19-year-old female with polycystic ovary syndrome who presented with headaches, pulsatile tinnitus, and transient visual obscurations; examination showed grade 1 papilledema. Neuroimaging demonstrated features of raised intracranial pressure (ICP) without mass lesion or venous sinus thrombosis, and lumbar puncture confirmed an elevated opening pressure of 39 cm H₂O. Blood tests revealed iron deficiency without anaemia (ferritin 21 µg/L, transferrin saturation 15%, hemoglobin [Hb] 121 g/L). Her symptoms improved after the lumbar puncture, and she was managed with iron supplementation and lifestyle advice for weight loss, with migraine prophylaxis also considered. This report highlights iron deficiency, even without overt anaemia, as a potential modifiable factor in IIH. Screening iron studies should be considered in young females presenting with IIH symptoms.

## Introduction

Idiopathic intracranial hypertension (IIH), also known as pseudotumor cerebri, is characterised by signs of raised intracranial pressure (ICP) such as headaches, papilledema, and transient visual disturbances, in the absence of an intracranial mass or hydrocephalus [1]. The condition often affects young women of childbearing age, particularly those who are overweight or obese [1]. In addition to obesity, several risk factors have been identified, including certain medications and endocrine disorders [2]. Recent studies have highlighted an association between iron deficiency anaemia (IDA) and IIH, even though the causal mechanisms remain uncertain [1,2]. Proposed explanations include increased blood viscosity leading to elevated cerebral venous pressure, and chronic hypoxia contributing to cerebral edema and impaired cerebrospinal fluid resorption [2].

Polycystic ovary syndrome (PCOS), a common endocrine disorder in young women, has also been reported more frequently among patients with IIH [3]. Both IIH and PCOS share risk factors such as obesity and insulin resistance, raising the possibility of overlapping pathophysiological pathways [3]. Weight management remains the only proven disease-modifying therapy for IIH, and it also benefits metabolic abnormalities seen in PCOS [4]. Iron deficiency without overt anaemia is common in young women and may impact neurological function even before anaemia develops [1,2]. We present the case of a young female with IIH in the context of PCOS and iron deficiency without anaemia, underscoring the importance of recognising iron deficiency as a potentially modifiable risk factor in IIH.

## Case presentation

A 19-year-old female with a history of PCOS presented with a one-month history of daily headaches and pulsatile tinnitus. The headaches were diffuse, pressure-like, and worse when lying down and on waking in the morning, sometimes severe enough to awaken her from sleep. She also reported transient episodes of blurred vision when standing quickly or bending forward, but denied persistent diplopia, seizures, or limb weakness. Two weeks before presentation, she had developed a constant “whooshing” sound in her right ear synchronous with her pulse and mild right-sided neck discomfort. She was not on any medications. She reported gaining 12 kilograms over the past two years. Her family history was unremarkable for neurological or haematological disorders.

Laboratory tests showed iron deficiency without anaemia: haemoglobin: 121 g/L, haematocrit: 0.36, mean corpuscular volume: 82 fL, serum iron: 11.0 µmol/L, transferrin saturation: 15%, and ferritin: 21 µg/L. Other haematological and biochemical tests, including thyroid function, B12, folate, and inflammatory markers, were normal. The results indicated reduced iron stores despite a haemoglobin level within the normal range. The full panel is summarised in Table 1.

**Table 1:** Laboratory investigations at presentation ^*^Outside the stated reference range

Parameters	Patient values	Reference range
Vitamin B12	304 ng/L	180–914 ng/L
Folate (serum)	2.5 µg/L^*^	3.1–20.0 µg/L
Ferritin	21 µg/L^*^	30–336 µg/L
C-reactive protein (CRP)	4.8 mg/L	0–5 mg/L
White cell count	11.91 ×10^9^/L^*^	3.7–11.0 ×10^9^/L
Haemoglobin (Hb)	121 g/L	120–165 g/L
Platelets	405 ×10^9^/L^*^	150–400 ×10^9^/L
Red blood cells (RBC)	4.20 ×10^12^/L	3.80–5.00 ×10^12^/L
Haematocrit	0.361 L/L	0.360–0.460 L/L
Mean corpuscular volume (MCV)	86.0 fL	83.0–101.0 fL
Mean corpuscular haemoglobin (MCH)	28.8 pg	27.0–32.0 pg
Mean corpuscular Hb concentration (MCHC)	335 g/L	315–360 g/L
Red cell distribution width (RDW)	13.70%	—
Platelet distribution width (PDW)	8.6 fL	—
Mean platelet volume (MPV)	8.7 fL^*^	9.0–13.0 fL
Neutrophils	8.6 ×10^9^/L^*^	1.7–7.5 ×10^9^/L
Lymphocytes	2.44 ×10^9^/L	0.9–4.0 ×10^9^/L
Monocytes	0.8 ×10^9^/L	0.2–1.0 ×10^9^/L
Eosinophils	0.04 ×10^9^/L	0.0–0.5 ×10^9^/L
Basophils	0.04 ×10^9^/L	0.0–0.1 ×10^9^/L
Nucleated RBC	0.00 ×10^9^/L	0.0–0.1 ×10^9^/L
Serum iron	11.0 µmol/L	11.0–32.0 µmol/L
Transferrin	3.0 g/L	1.7–3.6 g/L
Transferrin saturation	15%^*^	20–55%
Albumin	46 g/L	35–50 g/L
Alanine aminotransferase (ALT)	17 IU/L	<35 IU/L
Alkaline phosphatase (ALP)	52 IU/L	30–130 IU/L
Bilirubin (total)	9 µmol/L	0–21 µmol/L

On examination, the patient's blood pressure was 120/80 mmHg, and BMI was 29 kg/m². She was alert and oriented with normal cognition. Visual acuity was 20/20 bilaterally. Fundoscopy showed subtle bilateral optic disc swelling consistent with Frisén grade 1 papilloedema (no fundus photographs available at the time of assessment; findings described by the on-call ophthalmologist). No retinal haemorrhages were seen. Cranial nerve exam was normal, with no sixth nerve palsy and full extraocular movements. Motor and sensory function were intact, and coordination tests were normal. There was mild tenderness of the right side of her neck, but no nuchal rigidity. The rest of her general and neurological exam was normal. Given the clinical suspicion of raised intracranial pressure, a differential diagnosis of idiopathic intracranial hypertension versus secondary intracranial hypertension (e.g., venous sinus thrombosis, intracranial mass, or elevated central venous pressure) was considered.

**Figure 1:**
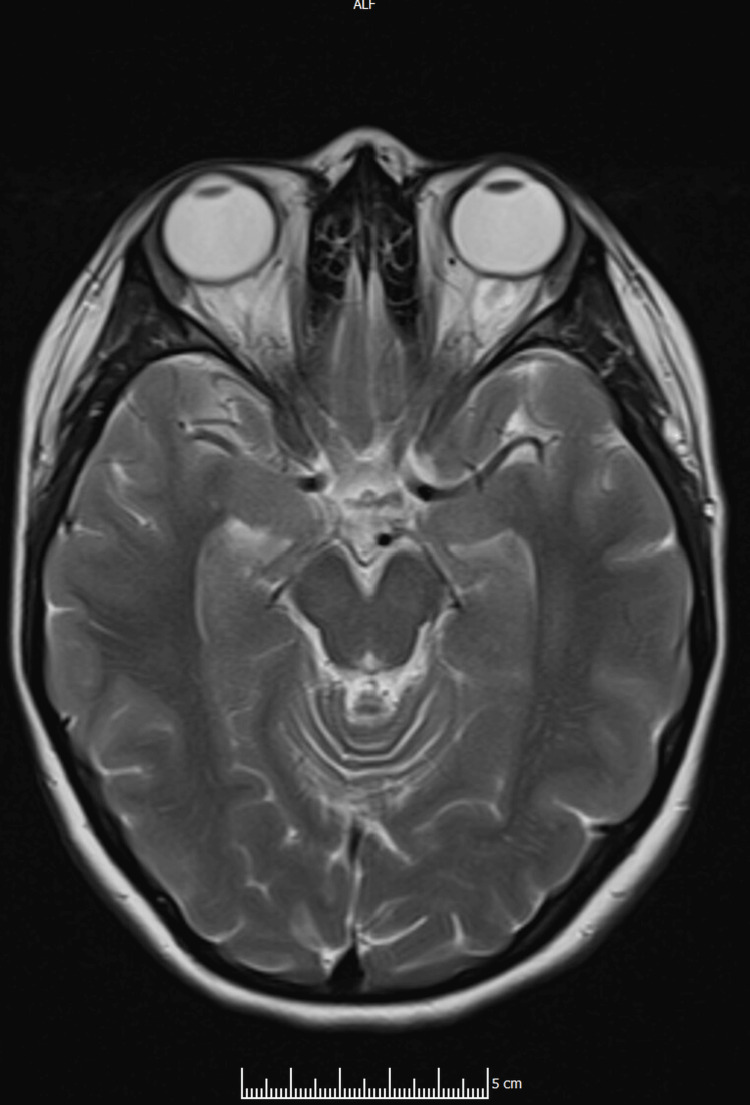
Axial orbital MRI (T2): posterior scleral flattening and perioptic subarachnoid space distension MRI: magnetic resonance imaging

**Figure 2:**
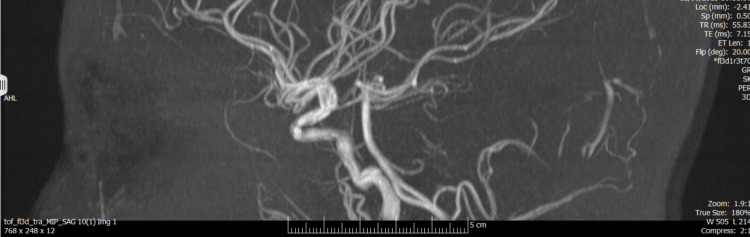
Time-of-flight MRA stills: normal intracranial arterial anatomy (sagittal section) MRA: magnetic resonance angiogram

**Figure 3:**
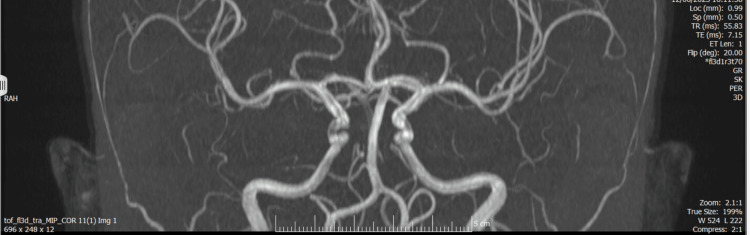
Time-of-flight MRA stills: normal intracranial arterial anatomy (coronal section) MRA: magnetic resonance angiogram

**Figure 4:**
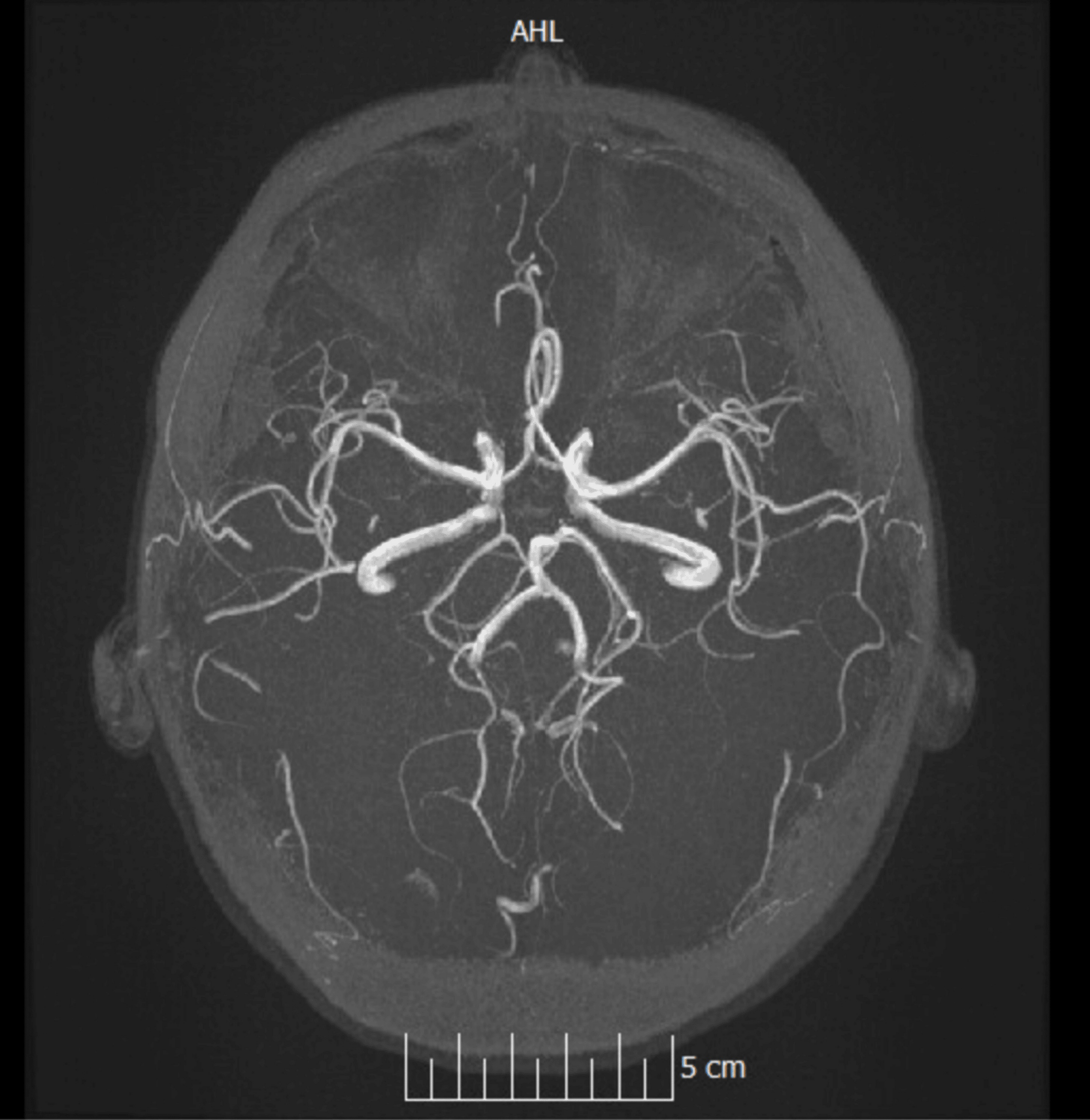
Time-of-flight MRA stills: normal intracranial arterial anatomy MRA: magnetic resonance angiogram

A non-contrast CT head (CTH) was obtained at presentation as part of the initial work-up (video S7 in the Appendices). MRI of the brain was subsequently performed (Figure 1), together with MR venography (MRV) and MR angiography (MRA) (Figures 2-4). Imaging showed no space-occupying lesion, hydrocephalus, or meningeal enhancement. However, features consistent with raised intracranial pressure were present: partially empty sella turcica, flattening of the posterior sclerae, and distension of the perioptic subarachnoid space (Figure 1). The venous sinuses were patent with no evidence of thrombosis, and MRA excluded arteriovenous malformations or dural fistulas. MRV is provided in supplementary videos S1 and S2 (Appendices); additional MRA rotating reconstructions are provided in supplementary videos S3-S5 (Appendices); the full MRI brain parenchyma cine is provided in supplementary video S6 (Appendices), which includes orbital levels illustrating posterior scleral flattening and perioptic subarachnoid space distension on axial views.

A lumbar puncture in the lateral decubitus position revealed an opening pressure of 39 cm H₂O (normal: <25 cm H₂O in adults). Cerebrospinal fluid (CSF) was clear, with normal protein and glucose levels, no pleocytosis, and negative cultures and cytology. Drainage of 20 mL of CSF lowered the closing pressure to 20 cm H₂O, resulting in symptomatic headache relief. These findings confirmed the diagnosis of IIH.

The patient was treated with an intravenous iron loading dose, followed by oral supplementation. She was advised to take vitamin C with her oral iron to enhance absorption. Given her BMI and recent weight gain, lifestyle modification aiming for a 15% reduction in body weight was recommended. Acetazolamide, the first-line pharmacological agent for IIH, was considered but deferred initially in light of her improvement after lumbar puncture and concurrent management of modifiable factors. Migraine prophylaxis with amitriptyline, propranolol, or candesartan was discussed, should her headaches persist. She was referred to ophthalmology for ongoing visual field monitoring and instructed to seek urgent review for any visual decline or recurrence of pulsatile tinnitus.

At the five-week follow-up, her headaches had improved markedly with weight reduction and iron supplementation alone, and hence prophylactic therapy was deferred.

## Discussion

IIH requires prompt recognition and targeted treatment to protect vision and relieve symptoms; standard care centres on ICP reduction alongside risk-factor modification [1]. Although IDA has been reported in association with IIH, it remains relatively uncommon. Since Praël’s first report in 1840, fewer than 100 such cases have been documented; in some reports, correction of anaemia alone has led to full resolution of IIH symptoms. The mechanism remains uncertain, though severe iron deficiency may induce a hyperviscous state of the blood via reactive thrombocytosis, thereby elevating venous sinus pressures without frank thrombosis, or may create a chronic hypoxic state that impairs cerebrospinal fluid absorption and contributes to cerebral oedema [2].

PCOS, which often coexists with obesity and insulin resistance, was also present in this patient. Several studies have noted higher-than-expected rates of PCOS in women with IIH. The overlap may reflect shared metabolic or hormonal factors rather than PCOS being an independent risk factor; importantly, recent prospective work suggests that comorbid PCOS does not necessarily worsen headache or vision outcomes in IIH, reinforcing the importance of targeting common risk factors such as obesity [3]. Obesity remains the most consistent modifiable risk factor for IIH, and even modest weight reduction of 5-15% has been shown to reduce ICP and improve papilledema [4].

The management of IIH focuses on lowering ICP to protect vision and alleviate symptoms. First-line approaches include acetazolamide to reduce CSF production and structured weight-loss strategies [4]. Therapeutic lumbar puncture can provide temporary relief by lowering CSF pressure [5]. Headache management is also a critical component of care: many IIH patients continue to experience disabling headaches even after ICP is controlled, and these often resemble migraine; in such cases, prophylactic therapy (e.g., amitriptyline, propranolol, or candesartan) may be beneficial [6,7]. For patients with progressive or severe disease, surgical options such as optic nerve sheath fenestration or CSF diversion may be required, and in selected cases, venous sinus stenting can be considered when focal transverse sinus stenosis with a significant pressure gradient is demonstrated [8]. This is supported by randomised trial evidence showing that bariatric surgery achieves greater ICP reduction than community weight-management programs [9]. Contemporary clinic-focused guidance is consistent with these strategies [10]. Recent systematic review data and a contemporary complications series further inform the evidence base and risks regarding venous sinus stenting [11,12].

In our patient, iron deficiency was present without anaemia, but it is plausible that even moderate reductions in iron stores contributed to elevated ICP. By correcting her iron status, we aimed to reduce this possible aggravating factor and to prevent progression to frank anaemia, which is itself a recognised risk factor for raised ICP [1,2]. This case underscores the importance of a thorough systemic evaluation in IIH: even when haemoglobin is normal, iron deficiency may be revealed by iron studies and represent a simple, effective adjunctive target within standard IIH care [1,2]. We therefore recommend checking iron studies in IIH patients regardless of whether typical risk factors are present, as treating iron deficiency or IDA can be a simple, effective adjunct to standard IIH care [1,2,6].

This report has a major limitation: it involves a single-patient experience and cannot establish causality between iron deficiency and raised ICP; observed improvement may partly reflect concurrent treatments, weight change, or the natural history of IIH. Hence, larger cohort studies and prospective trials are needed to clarify the mechanisms and quantify the independent effect of iron repletion on visual and headache outcomes.

## Conclusions

IIH requires prompt recognition to protect vision. In young women, iron deficiency may coexist even when haemoglobin is normal and should be assessed routinely alongside imaging and lumbar puncture, as treating confirmed deficiency is a low-risk adjunct to standard care. Weight loss remains disease-modifying and should be prioritised, with acetazolamide considered to lower CSF production when indicated and ophthalmic monitoring guiding escalation to surgical options if vision is threatened. Incorporating iron studies at presentation can prevent missed deficiency and may improve patient outcomes.

## Appendices

Supplementary videos

Video S1. MR Venography (MRV) - Projection 1

Video S2. MRV - Projection 2

Video S3. TOF-MRA - Rotating MIP

Video S4. MRA Demonstrating the Normal Circle of Willis

Video S5. TOF-MRA - Further Projections Corroborating Normal Arterial Anatomy

Video S6. MRI Brain Parenchyma

*Video S7. Non-Contrast CT Head (CTH) at Presentation*
 
